# Connecting the dots: Path model to identify key phenotypic traits for screening plants with tolerance to nitrogen deficiency

**DOI:** 10.1371/journal.pone.0288729

**Published:** 2023-08-18

**Authors:** Ranjeeta Adhikari, Krishna Nemali

**Affiliations:** 1 Department of Horticulture and Landscape Architecture, Purdue University, 625 Agriculture Mall Drive, West Lafayette, IN, United States of America; 2 Bayer Crop Science, Soda Springs, ID, United States of America; University of Brescia: Universita degli Studi di Brescia, ITALY

## Abstract

Varieties that tolerate low nitrogen (N) application rates can reduce fertilizer costs, minimize nitrate leaching and runoff losses, and lower overall CO_2_ emissions associated with fertilizer manufacturing. The goal of our research is to show the usefulness of path models to identify key phenotypic traits for screening plants with a tolerance to low N application rates. We grew tolerant and sensitive cultivars of poinsettia (*Euphorbia pulcherrima*) using a water-soluble fertilizer (15-5-15 Cal Mag) in both optimal (electrical conductivity of 2.5 dS·m^-1^) and N-deficient (electrical conductivity of 0.75 dS·m^-^1) treatments and measured 24 different traits at the cellular, leaf, and whole-plant scales in both cultivars and treatments. The experiment was laid out as a split-plot design with N treatments as main plots and cultivars as sub-plots, with five replications. Path analysis was conducted to develop sequential relationships among these traits. Statistical comparisons between tolerant and sensitive cultivars in the N-deficient treatment indicated an increase in shoot biomass (19.9 vs 14.4 g), leaf area (2775 vs 1824 cm^2^), leaf dry weight (14.7 vs 10.0 g), lateral root dry weight (3.7 vs 2.4 g), light-saturated photosynthesis (14.5 vs 10.1 μmol∙m^-2^∙s^-1^), maximum electron transport rate (119 vs 89 μmol∙m^-2^∙s^-1^), chlorophyll content (28.1 vs 12.9 g∙100g^-1^), leaf N content (27.5 vs 19.9 mg∙g^-1^), and fine root N content (26.1 vs 20.9 mg∙g^-1^), and a decrease in anthocyanin content (0.07 vs 0.16 ΔOD∙g^-1^). The path model indicated that an increase in the lateral root growth and fine root N content can lead to an increase in the leaf N content, in the N-deficient treatment. There were three separate paths that connected higher leaf N content to increased shoot biomass. These paths were mediated by the levels of anthocyanin, chlorophylls, and light-saturated photosynthesis rate (or rubisco capacity). The light-saturated photosynthesis model suggested that the increased uptake of N by fine roots in the tolerant cultivar was likely supported by the photosynthates translocated from the shoot to the root. Leaf N content was associated with multiple plant responses in the N-deficient treatment, and can be a useful screening trait for developing new cultivars, especially in marker-assisted molecular breeding.

## Introduction

Most of the N fertilizers are manufactured from ammonia synthesized in the Haber Bosch process. This process consumes a significant amount of energy (using natural gas and coal) and is responsible for more than 1.2% of global CO2 emissions [1]. Recent increases in energy costs, especially that of natural gas, have significantly increased the cost of N fertilizers (US $235/mt in April 2020 to US $925/mt in April 2022; Source: World Bank). Reducing N application to crops may lead to lower production costs and the carbon footprint; however, N is essential for the growth and development of crops [2] and lower N application can result in reduced growth [3, 4] with significant economic losses to growers [5]. A more practical approach to reducing overall N application is to develop varieties that can tolerate low rates of N application during production. Molecular breeding is one of the popular methods to develop new crop varieties. This method requires identifying key trait(s) and genetic markers related to key traits. Once genetic markers are developed, thousands of plants can be screened rapidly using high-throughput genotyping methods [6] for the presence of a trait (i.e., without actually screening for the phenotype). Although a large volume of information is known about physiological traits in plants that are affected by N, limited information exists on key traits with a large influence on tolerance to low N application rates. Such information can increase the overall soundness of crop improvement programs.

Knowledge of the growth and development responses in plants to N supply is essential to develop key traits. Plants exposed to N- deficient conditions can respond by increasing N-uptake from the substrate and/or N-utilization in metabolic processes. To increase N-uptake efficiency under N-deficient conditions, plants can specifically increase root growth to explore more soil volume [7–9]. An increase in root-to-shoot ratio (i.e., a ratio of root biomass to shoot biomass) was observed in plants exposed to N- deficiency [10]. Plants can increase the lengths of both primary and lateral roots [11] to explore deeper and larger soil volumes. In addition, an increase in the number of fine roots was observed in plants [12], likely to increase nutrient absorption. Plants with increased N-utilization efficiency invest more N in chlorophyll and rubisco [13]. Nitrogen is a component of the chlorophyll molecule [14], a pigment involved in the light absorption process of photosynthesis. A majority of leaf N is present in rubisco [14] and increased levels of leaf N can increase rubisco activity [15, 16]. In addition, N is a component of thylakoid proteins, cytochrome complex, and ATP synthase, all of which are involved in the electron transport process of photosynthesis [17]. Given this, a strong positive correlation exists between the rate of photosynthesis and leaf N status in plants [15, 18, 19].

The goal of the present research is to show the usefulness of path models to identify key traits in plants associated with tolerance to low N application rates. We used poinsettia in our research due to its economic importance as a holiday ornamental plant and high N demand/ sensitivity during production. The objectives of this study were to (i) measure and compare physiological traits at the cellular, leaf, and whole-plant scales in sensitive and tolerant poinsettia cultivars exposed to both optimal and N-deficient conditions, (ii) develop statistical path models that sequentially connect the physiological traits at different scales in the N-deficient treatment, and (iii). identify key trait(s) which directly influence multiple traits with positive effects on tolerance to low N application rates.

## Materials and methods

### Plant materials

Cuttings of poinsettia (*Euphorbia pulcherrima*) cultivars (Jubilee Red and Peterstar Red) were obtained from Dummen Orange (Columbus, OH, USA). Based on the producer catalog information, Jubilee Red is dark green in appearance with heat and cold stress tolerance than Peterstar Red. Therefore, Jubilee Red and Peterstar Red were treated as tolerant and sensitive cultivars respectively in the study. The cuttings were dipped in 0.1% indole butyric acid rooting hormone (Rhizopon® AA #, Earth City, MO, US) and inserted in 48-cell-packs (Greenhouse Mega store, Danville, IL, USA) filled with propagation mix (Berger BM2 germination mix, Berger, Quebec, Canada). Rooted cuttings were transplanted into 6-inch containers (1.33 L, Greenhouse Megastore, Danville, IL, USA) filled with a peat-based substrate (Berger BM8 growing mix, Berger, Quebec, Canada).

### Fertigation system

Plants were grown in a custom-built ebb-flow system containing low-rise flood tables (1.22 m × 1.22 m, SKU # AALR44B, Active Aqua, Hydro Farm, CA, USA), reservoirs (151.4 L, SKU#HGRES40K Active Aqua, Hydro Farm, CA, USA), flexible poly-vinyl tubing (1.9 cm ID, model # 714565, Everbilt Co., Home Depot, Atlanta, GA, USA), and submersible pumps (18.9 L∙min^-1^, Item # 52216/ MD11300, Total Pond, West Palm Beach, FL, USA). An irrigation timer (Titan controls Apollo 6, Part #734110, Hawthorne Gardening Company, Vancouver, WA, USA) was used to turn on the pumps every day for a period of 15 min during which the level of solution in the flood table raised by approximately 3 cm for substrate in the containers to absorb the solution by capillarity. The solution drained back into the reservoir after the pumps were turned off through the outlet of the flood table (S1 Fig).

### Environmental conditions

The experiment was conducted in a glass-covered greenhouse maintained at a daily light integral of 12.1 ± 0.84 mol∙m^-2^ and an average day/night temperature of 25.4 ± 1.22/ 20.8 ± 0.91°C during the experiment. Supplemental lighting was provided using high-pressure sodium lamps to maintain optimal light intensity and long-day photoperiod of 18 hours. The photosynthetic photon flux density (*PPFD;* intensity of light between 400 to 700 nm) was measured using four quantum sensors (Li-190 R, Li-COR, Lincoln, NE, USA) and air temperature was measured using thermistors (ST-100, Apogee Instruments, Inc., Logan, UT, USA) connected to a datalogger (CR 1000, Campbell Scientific, Logan, UT, USA).

### Treatments

Plants were grown in N-deficient (solution electrical conductivity or EC level of 0.75 dS·m^-1^; equivalent to a N concentration of 50 mg∙L^-1^) and optimal (solution EC level of 2.5 dS·m^-1^; equivalent to a N concentration of 150 mg∙L^-1^) conditions using a water-soluble fertilizer containing 15N: 2.2P: 12.5K (15-5-15 Cal Mg, Peter’s Excel, ICL Specialty fertilizer, UK). A dielectric sensor (5TE, Meter group, WA, US) was used to measure EC and prepare fertilizer solutions. Fresh fertilizer solution was supplied on one day of the week and tap water was provided to plants during the remaining days. The pH of the fertilizer solution was maintained between 5.6 to 6.8 during the experiment.

### Measurements and calculation

*(i)*. *Cellular scale*. Total chlorophyll (CHL), carotenoid (CTD), and anthocyanin (ACN) content of the leaves were measured in the laboratory using Biomate 160 UV-VIS spectrophotometer (Thermo Fischer Scientific, Waltham, MA) as described by [20]. The CHL-a, CHL-b, and CTD concentrations (μg∙ mL^-1^) were measured using absorbance values as follows:

CHL−a=12.25×A663.2−2.79×A646.8

CHL−b=21.5×A646.8−5.1×A663.2

$$CTD=\frac{1000\times A470-1.8\times Chl\;a-85.02\times Chl\;b}{198}$$

where, A663.2, A646.8, and A470 were absorbance values at 663.2, 646.8, and 470 nm, respectively. The pigment content on a fresh weight basis (mg∙100g^-1^) was calculated by multiplying concentration with extraction volume and dilution factor, and dividing by the sample weight. The CHL was calculated by adding the contents of CHL-a and CHL-b.

Relative ACN concentration (ΔOD∙g^-1^) was measured as the difference in absorbance at 530 and 600 nm.

ACN=A530−A600

where, A530 and A600 represent absorbance at 530 and 600 nm, respectively.

The N content (mg∙g^-1^) of leaves (N_Leaf_), stem (N_Stem_), lateral roots (N_LR_), and fine roots (N_FR_) was measured using the Dumas method in an analytical laboratory (A & L Great Lakes, IN, USA). Different tissue samples were dried separately in a forced air oven and powdered before analyzing in the laboratory.

*(ii)*. *Leaf scale*. Plant photosynthesis response to varying *PPFD* was measured using Li-COR 6400XT portable photosynthesis system (Li-COR Biosciences, Lincoln, Nebraska, USA). Measurements were taken on a fully expanded leaf during the daytime and on a sunny day. The levels of CO_2_, leaf temperature, and relative humidity inside the leaf cuvette were maintained at 400 μmol·mol^-1^, 28°C, and 40–60%, respectively. Photosynthesis was measured at varying *PPFD* levels of 1500, 1000, 800, 600, 400, 200, 150, 100, 50, and 0 μmol·m^-2^·s^-1^. The light levels were maintained by a built-in LED (10% B and 90% R) light source inside the leaf cuvette. Each light level was maintained for approx. 4–5 min until the fluxes of CO_2_ and water vapor, and rate of photosynthesis were stable before recording measurements. Maximum gross photosynthetic rate (P_g,max_), light-use efficiency (LUE), and rate of dark respiration (R_d_) were estimated by fitting an exponential model to the measured data.

$$P_{n}=P_{gmax}[1-\exp^{\left(-\frac{\alpha \times I}{\mathrm{Pgmax}}\right)}]-R_{d}$$

where, P_n_ is net photosynthetic rate (μmol CO_2_·m^-2^·s^-1^), α is LUE (μmol CO_2_·mol^-1^), *I* is *PPFD* (μmol·m^-2^·s^-1^), P_g,max_ is maximum gross photosynthesis (μmol CO_2_·m^-2^·s^-1^), and R_d_ is rate of dark respiration (μmolCO_2_·m^-2^·s^-1^). Light saturation point (LSP, (μmol·m^-2^·s^-1^) was calculated from the above equation by assuming P_n_ equal to 0.85 P_g,max_ and solving the equation for LSP [21].

For measuring the relation between photosynthesis and intercellular CO_2_ concentration, Li-COR 6400XT attached to a fluorometer was used. The temperature, relative humidity, and *PPFD* inside the cuvette were maintained at 28°C, 40–60%, and 1000 μmol·m^-2^·s^-1^ respectively. A steady-state photosynthesis rate and chlorophyll fluorescence parameters were measured at an ambient CO_2_ concentration of 400 μmol·mol^-1^. Then photosynthesis rate and chlorophyll fluorescence parameters were measured at varying levels of CO_2_ concentrations of 400, 600, 800, 1000, 600, 400, 200, 150, 100, and 50 μmol·mol^-1^ in the leaf cuvette. At each level of CO_2_ concentration, the measurements took approximately 4–5 minutes to reach stable values. The parameters such as V_cmax_ (maximum carboxylation rate, μmol CO_2_·m^-2^·s^-1^), J_max_ (maximum electron transport rate, μmol electrons·m^-2^·s^-1^), and g_m_ (mesophyll conductance to CO_2_, mol·m^2^·s^-1^) were calculated as described by [22]:

The value of V_cmax_ was calculated as the slope of the linear relationship between A and fˈ:

$$A=f^{\prime }V_{cmax}+c$$

The values of *fˈ* were calculated as:

$$f\prime =\frac{C_{i}-\Gamma^{*}}{C_{i}+K_{c}(1+\frac{O}{K_{o}})}$$

where, C_i_ is leaf internal CO_2_ concentration (selected range: 50 to 200 μmol∙mol^-1^), Γ* is CO_2_ compensation point in the absence of dark respiration (31 μmol∙ mol^-1^ at 25°C, as described by [23]), and K_c_ and K_o_ are the Michaelis-Menten coefficients of Rubisco activity for CO_2_ and O_2_ calculated from equations:

$$K_{c}={exp}^{\left(38.05-\frac{79.43}{R(T_{1}+273.15)}\right)}\;\mathrm{and}\;K_{o}={exp}^{\left(20.30-\frac{36.38}{R(T_{1}+273.15)}\right)}$$

where, T_1_ is leaf temperature (°C), and R is the molar gas constant (R = 8.315 J·mol^-1^·K^-1^).

The value of J_max_ was calculated as the slope of the linear relationship between A and gˈ:

$$A=g\prime J_{max}$$

The value of gˈ was calculated as:

$$g\prime =\frac{C_{i}-\Gamma^{*}}{{4.5C}_{i}+10.5\Gamma^{*}}$$

where, C_i_ is leaf internal CO_2_ concentration (selected range: 200 to 700 μmol∙mol^-1^) and Γ* is CO_2_ compensation point in the absence of dark respiration (31 μmol∙ mol^-1^ at 25°C).

Mesophyll conductance (g_m_) was calculated by using variable J method as:

$$g_{m}=\frac{A}{C_{i}-\frac{\Gamma^{*}(J+8(A+R_{d}))}{\left(J-4(A+R_{d})\right)}}$$

where, A, C_i_, and J are photosynthesis rate, leaf internal CO_2_ concentration, and electron transport rate at an ambient CO_2_ concentration of 400 μmol∙ mol^-1^ respectively, Γ* is CO_2_ compensation point in the absence of dark respiration (31 μmol∙ mol^-1^ at 25°C), and R_d_ is respiration rate in light measured as the y-intercept of the relationship between A and *fˈ*.

*(iii)*. *Whole-plant scale*. Plants were harvested 45 days after transplanting and leaves were separated from stem. Leaf area (Area_Leaf_, cm^2^) was measured using a leaf area meter (Li-3100C, Li-COR, Biosciences). After washing, lateral roots and fine roots (small roots originating from lateral roots) were separated. Leaves, stem, lateral roots, and fine root samples were dried separately in a forced dry oven at 70°C before measuring leaf dry weight (DW_Leaf_), stem dry weight (DW_Stem_), lateral root dry weight (DW_LR_), and fine root dry weight (DW_FR_). Shoot dry weight (DW_Shoot_) was calculated by adding DW_Leaf_ and DW_Stem_, and root dry weight (DW_Root_) as the sum of DW_LR_ and DW_FR_. Specific leaf area (SLA, cm^2^∙g^-1^) was calculated as the ratio of Area_Leaf_ to DW_Leaf_. Root weight ratio (RWR, g∙g^-1^) was calculated as DW_Root_ to total dry weight (DW_Shoot_ + DW_Root_).

### Experimental design and statistical analyses

The experiment used a split-plot design with N treatments as main-plots and cultivars as sub-plots. There were five replications of main plot. The effects of fertilizer treatment and cultivar were analyzed using Proc GLIMMIX procedure of statistical analysis software (SAS 9.4, Cary, NC, USA). Least square means were separated using Tukey’s honestly significant difference (HSD). Exponential equations were fitted to photosynthesis-*PPFD* and photosynthesis-C_i_ response curves using Proc NLIN procedure of SAS. As path analysis allows for exploring direct and indirect associations simultaneously among a set of observed variables, it was used to test the hypothetical relationships among variables at different scales in the N-deficient treatment. Further the path analysis can provide slope value of the relationship between two variables and error associated with predicting one variable from the other within a path. The path analysis for traits was conducted using Proc CALIS procedure of SAS. A chi-square analysis was used to test whether the hypothetical path model was a good fit to the observed data. A lack of significance chi square value indicates that model is a good fit to the observed data. For all statistical procedures and comparisons, *P* ≤ 0.05 was considered statistically significant.

## Results

### Tissue N, CHL, CTD, and ACN Levels

A significant interaction between N treatment and cultivar was seen for N_Leaf_ and N_FR_ (Table 1). No differences in N_Leaf_ and N_FR_ were observed between the cultivars in the optimal but significantly higher levels were observed in the tolerant than sensitive cultivar in the N-deficient treatment. The N_Stem_ and N_LR_ were not different between the cultivars in both treatments, but the values were higher in the optimal than N-deficient treatment. Further, results indicated a significant interaction effect between N treatment and cultivar on ACN, CHL, and CTD levels (Table 1). The ACN levels were higher in the sensitive than tolerant cultivar in the N-deficient, whereas there were no differences in ACN levels between cultivars in the optimal treatment. The levels of CHL and CTD were not different between the cultivars in the optimal treatment but the values were significantly higher in the tolerant than sensitive cultivar in the N-deficient treatment.

**Table 1:** Differences in traits at cellular scale, including leaf nitrogen content (N_Leaf_), stem nitrogen content (N_Stem_), lateral root nitrogen content (N_LR_), fine root nitrogen content (N_FR_), anthocyanin content (ACN), carotenoid content (CTD), and total chlorophylls content (CHL), between sensitive and tolerant cultivars exposed to nitrogen deficient and optimal conditions. Least square means followed by different letters are statistically different (*P* ≤ 0.05).

Trait	Deficient		Optimal	
	Sensitive	Tolerant	Sensitive	Tolerant
N_Leaf_ (mg∙g^-1^)	19.9 c	27.5 b	36.9 a	38.7 a
N_Stem_ (mg∙g^-1^)	12.2 b	14.4 b	28.0 a	30.1 a
N_LR_ (mg∙g^-1^)	13.7 b	13.2 b	19.0 a	19.8 a
N_FR_ (mg∙g^-1^)	20.9 c	26.1 b	35.6 a	35.6 a
ACN (ΔOD∙g^-1^)	0.162 a	0.074 b	0.016 c	0.018 c
CTD (mg∙100g^-1^)	1.21 b	4.80 a	3.79 a	5.35 a
CHL (mg∙100g^-1^)	12.95 b	28.11 a	27.78 a	33.48 a

### Leaf photosynthesis responses and photosynthetic parameters

**Fig 1:**
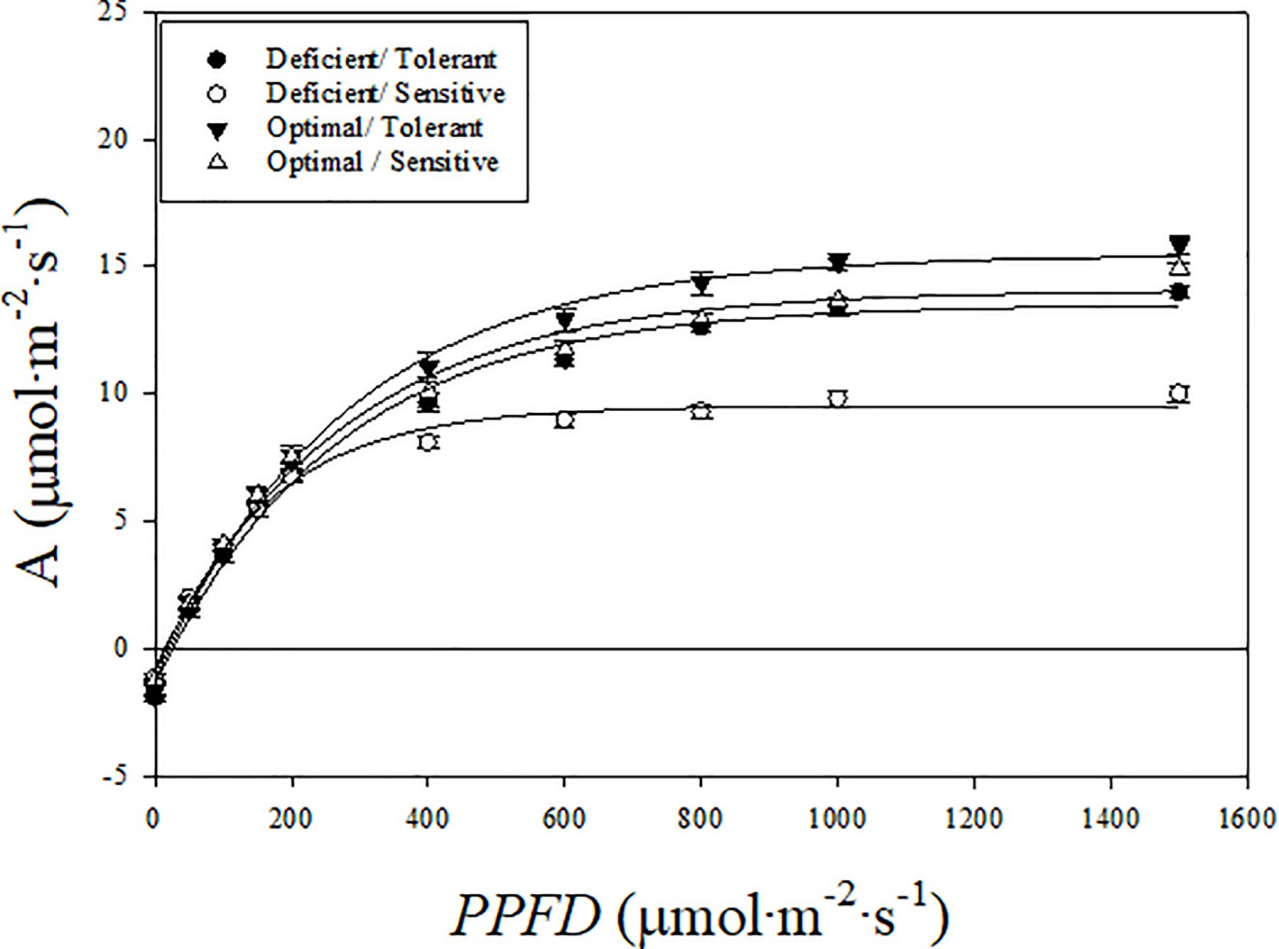
Photosynthesis (A) response to varying levels of photosynthetic photon flux density (*PPFD*) in tolerant and sensitive poinsettia cultivars grown in optimal and nitrogen deficient conditions. Mean photosynthesis and standard error (n = 5) at each *PPFD* level are shown.

The photosynthesis asymptotically increased to a maximum value with increasing *PPFD* in both cultivars and treatments (Fig 1). The net photosynthetic rate increased rapidly up to a *PPFD* of 400 μmol·m^-2^·s^-1^, gradually between 400 and 600 μmol·m^-2^·s^-1^ and very little with further increase in *PPFD*. The photosynthesis-*PPFD* response curves for both cultivars were not different in the optimal treatment, but they were different for cultivars in the N-deficient treatment. The curves in the N- deficient treatment started to separate between the tolerant and sensitive cultivars at a *PPFD* level of approximately 250 μmol·m^-2^·s^-1^, with higher levels of A observed in the tolerant than sensitive cultivar with increasing *PPFD* levels.

**Fig 2:**
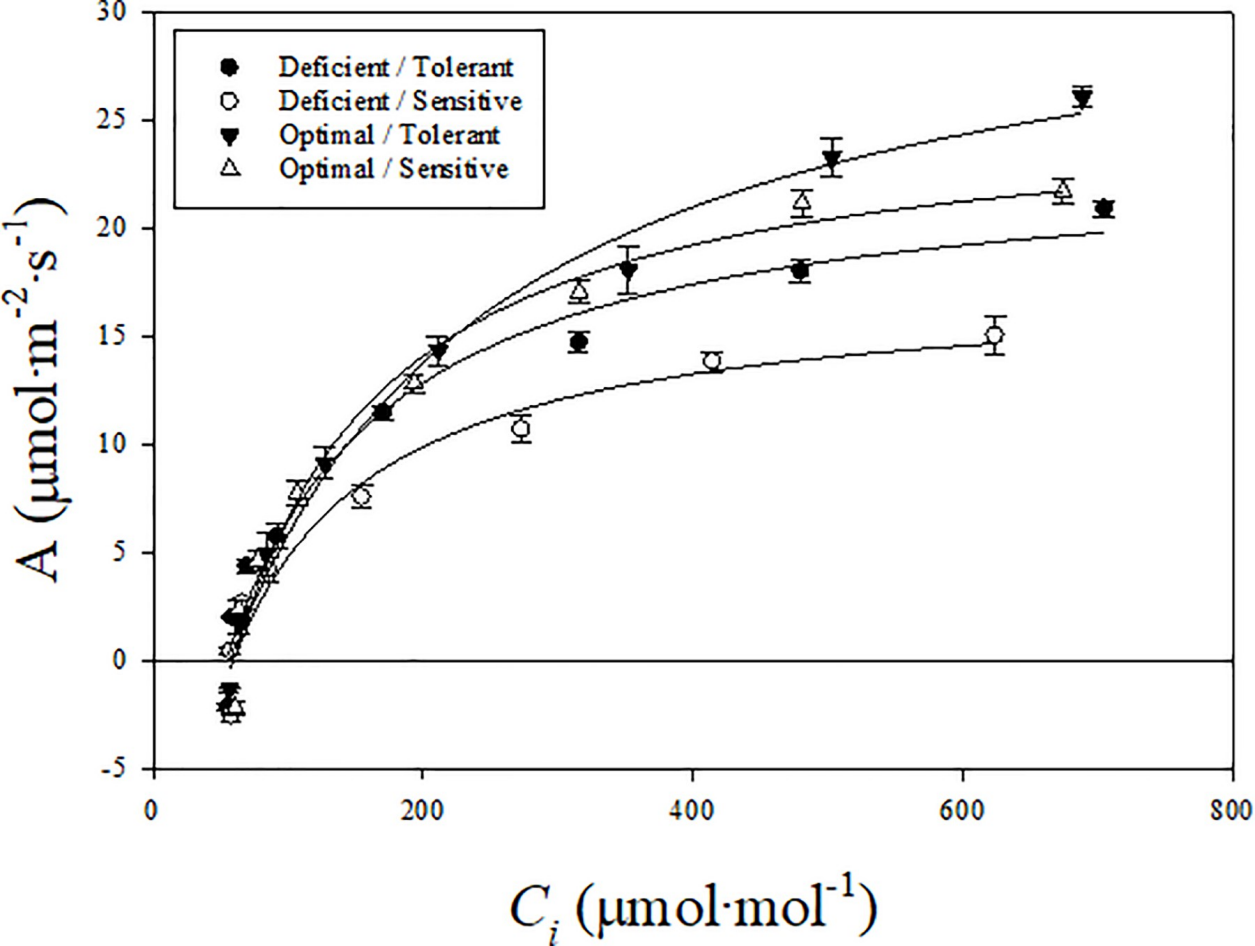
Photosynthesis (A) response to varying levels of intercellular carbon dioxide concentration (Ci) in tolerant and sensitive poinsettia cultivars grown in optimal and nitrogen deficient conditions. Mean photosynthesis and standard error (n = 5) at each Ci level are shown.

The photosynthesis rate increased asymptotically with increasing intercellular CO_2_ in both cultivars and N treatments (Fig 2). However, photosynthesis did not appear saturated at high levels of C_i_ in both cultivars and N treatments. In the optimal treatment, cultivars did not show differences in photosynthesis at any level of C_i_. However, in the N-deficient treatment, photosynthesis differences between the cultivars started to appear at low C_i_ and became larger with increasing C_i_ levels. Photosynthesis rate was higher in the tolerant than sensitive cultivar at a range of Ci levels in the N-deficient treatment.

The photosynthetic parameters such as R_d_, LCP, and LUE were not different between the cultivars in both treatments (Table 2). However, parameters measured at high *PPFD* levels such as LSP and P_g,max_ were higher in the tolerant than sensitive cultivar when exposed to N deficient conditions. Interestingly, P_g,max_ was higher in the tolerant than sensitive cultivar even in the optimal treatment. Both V_cmax_ and g_m_ were not different between the two cultivars in optimal and N-deficient treatments (Table 2). However, J_max_ was higher in the tolerant than sensitive cultivar when exposed to N-deficient conditions. There were no differences in J_max_ between the two cultivars in the optimal treatment.

**Table 2:** Differences in traits at leaf scale, including dark respiration rate (R_d_), maximum gross photosynthesis (P_g,max_), light compensation point (LCP), light-use-efficiency (LUE), and light saturation point (LSP) estimated from photosynthesis-light response (*A-PPFD*) curves, and maximum carboxylation rate (Vc_max_), maximum electron transport rate (J_max_), and mesophyll conductance (g_m_) estimated from photosynthesis-leaf internal carbon dioxide concentration response (*A-C*_*i*_) curves, between sensitive and tolerant cultivars exposed to nitrogen deficient and optimal conditions. Least square means followed by different letters are statistically different (*P* ≤ 0.05).

Trait	Deficient			Optimal		
		Sensitive	Tolerant		Sensitive	Tolerant
***A-PPFD parameters***						
R_d_ (μmol·m^2^·s^-1^)		-0.99 a	-1.36 a		-0.69 a	-1.20 a
P_g,max_ (μmol·m^2^·s^-1^)		10.10 c	14.47 b		15.53 b	17.41 a
LCP (μmol·m^2^·s^-1^)		19.3 ab	29.0 a		12.5 b	18.2 ab
LUE (μmol·mol^-1^)		0.064 a	0.059 a		0.064 a	0.060 a
LSP (μmol·m^2^·s^-1^)		375.2 b	569.2 a		536.2 a	585.2 a
***A-C***_***i***_ ***parameters***						
Vc_max_ (μmol·m^2^·s^-1^)		68.42 a	100.20 a		108.02 a	117.78 a
J_max_ (μmol·m^2^·s^-1^)		88.28 c	118.80 b		131.40 ab	147.60 a
g_m_ (mol·m^2^·s^-1^)		0.158 a	0.230 a		0.282 a	0.250 a

### Shoot and root growth responses

**Fig 3:**
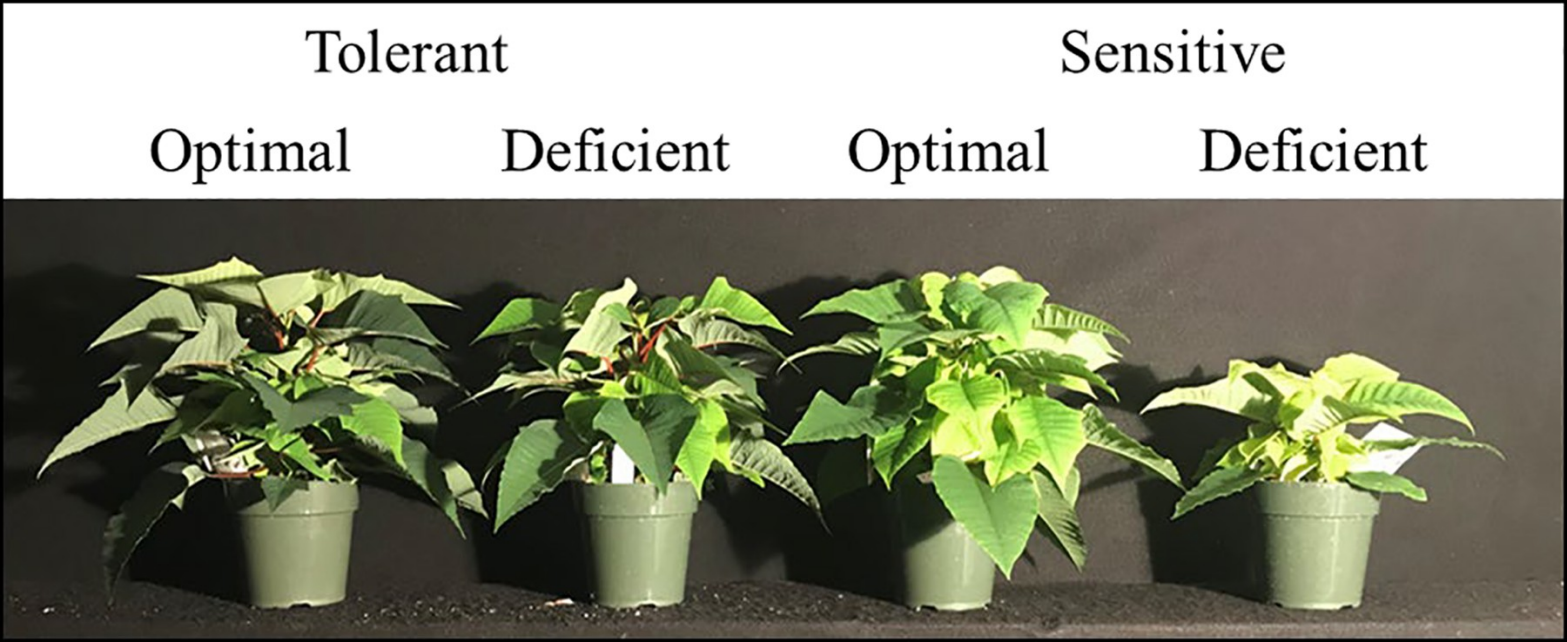
Representative poinsettia plants belonging to tolerant and sensitive cultivars grown in optimal and nitrogen deficient treatments.

The interaction between N treatment and cultivar was found to be significant for DW_Shoot_, DW_Leaf_, and Area_Leaf_ (Fig 3, Table 3). There was no difference in DW_Shoot_, DW_Leaf_, and Area_Leaf_ between the cultivars in the optimal treatment, whereas a significantly higher values of these measurements were seen in the tolerant compared to sensitive cultivar when exposed to N-deficient conditions. There were no differences in SLA between the cultivars in both treatments. Although DW_Root_ was not significantly different between the cultivars in both treatments, DW_LR_ was significantly higher in the tolerant than sensitive cultivar, when exposed to N-deficient conditions (Table 3). However, the differences in DW_FR_ and RWR were not significantly different between the two cultivars in both N treatments.

**Table 3:** Differences in traits at whole-plant scale, including shoot dry weight (DW_Shoot_), stem dry weight (DW_Stem_), leaf dry weight (DW_Leaf_), root dry weight (DW_Root_), lateral root dry weight (DW_LR_), fine root dry weight (DW_FR_), leaf area (Area_Leaf_), specific leaf area (SLA), and root weight ratio (RWR), between sensitive and tolerant cultivars exposed to nitrogen deficient and optimal conditions. Least square means followed by different letters are statistically different (*P* ≤ 0.05).

Trait	Deficient		Optimal	
	Sensitive	Tolerant	Sensitive	Tolerant
DW_Shoot_ (g)	14.38 b	19.93 a	20.26 a	20.95 a
DW_Stem_ (g)	4.37 a	5.14 a	5.48 a	4.77 a
DW_Leaf_ (g)	9.96 b	14.74 a	15.016 a	16.41 a
DW_Root_ (g)	4.01 a	5.33 a	4.16 a	4.15 a
DW_LR_ (g)	2.37 b	3.72 a	2.48 ab	2.79 ab
DW_FR_ (g)	1.59 a	1.55 a	1.74 a	1.41 a
Area_Leaf_ (cm^2^)	1824.7 c	2775.1 b	3756.9 a	3703.4 a
SLA (cm^2^∙g^-1^)	178.2 a	186.2 a	234.1 a	209.6 a
RWR (g∙g^-1^)	0.206 a	0.204 a	0.159 a	0.154 a

### Path models

**Fig 4:**
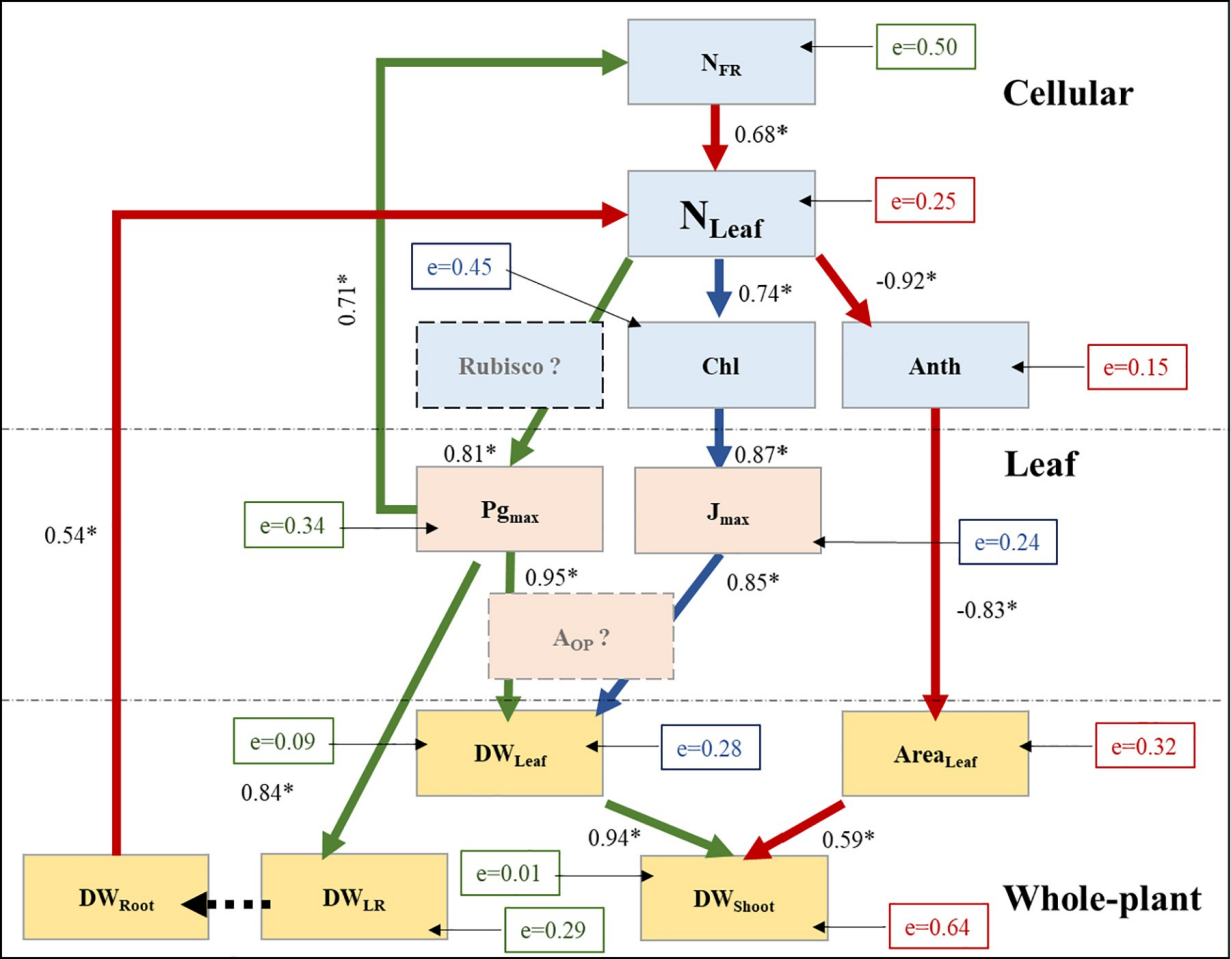
Path models showing relations among traits at cellular, leaf, and whole-plant scales in the nitrogen deficient treatment. Refer to Tables 1–3 for trait names. Paths are separated using different colored arrows. Numbers adjacent to connecting arrows with an asterisk indicate slope of the relationship between two variables and statistical difference from zero (*P* ≤ 0.05). Error variance associated with predicting a variable is shown in boxes with letter ‘e’. The box colors were matched with different path models. A black dashed arrow indicates possible relationship not significant in the model. Dashed boxes [Rubisco and A_OP_ (operating photosynthesis rate)] include possible connecting traits that were not measured in the study.

The inter-relations among the traits at the cellular, leaf, and whole-plant scales could be explained by three different path models (Fig 4). The *P-*values associated with chi-squares for each path was not significant (*P*>0.05), indicating that the models are a good fit to the data (i.e., null hypothesis that no difference exists between the observed values and predicted model is accepted). The first path (or ACN model; Fig 4 brown arrows) predicted that both DW_Root_ and N_FR_ positively affected N_Leaf_ in the N-deficient treatment. The model indicated that both N_Leaf_ and Area_Leaf_ were negatively related to ACN. These relations indicate that ACN levels increased with decreasing N_Leaf_ and larger levels of ACN in plants resulted in a decrease in Area_Leaf_. As expected, there were positive relations between Area_Leaf_ and DW_Shoot_ in the N-deficient conditions. A second path (CHL model; Fig 4, blue arrows) predicted that N_Leaf_ and CHL were positively related to each other. Further, the path predicted that CHL positively affected J_max_, which further positively influenced DW_Leaf_ in the N-deficient treatment. The third path (P_g,max_ model; Fig 4 green arrows) predicted that N_Leaf_ and P_g,max_ were positively related, and that P_g,max_ was positively associated with DW_LR_, DW_Leaf_, and N_FR_. Further, a positive relation was observed between DW_Leaf_ and DW_Shoot_. The error or ‘e’ values in all three models varied between 0.01 and 0.64 among traits. The lower error values indicate higher variance accounted by the predictor variable and *vice versa*. The error variance was low for traits such as N_Leaf_, ACN, J_max_, DW_Leaf_, and DW_Shoot_, moderate for traits such as N_FR_, CHL, and DW_Shoot_ (estimated from Area_Leaf_). It is likely that other predictor variables not included in the model affected those dependent variables with high error values.

## Discussion

In our study, there were no statical differences between the tolerant and sensitive cultivars in the optimal treatment for any trait except for P_g,max_. In the optimal treatment, P_g,max_ was lower by 11% in the sensitive than tolerant cultivar (Table 2). However, P_g,max_ decreased by 30% in the sensitive than tolerant cultivar in the N-deficient treatment. The larger difference in P_g,max_ between the sensitive and tolerant cultivars in the N-deficient compared to optimal treatment indicates a differential response in P_g,max_ to N-deficient conditions in the sensitive cultivar. Therefore, traits that were statistically different between the tolerant and sensitive cultivars were exhibited specifically in the N-deficient treatment.

In total, we measured 24 distinct traits from cellular, leaf, and whole-plant scales in our study. Of these, 50% of the traits were found to be different between the tolerant and sensitive cultivars in the N deficient treatment (Table 4). In general, traits at the cellular scale were mostly different between the tolerant and sensitive cultivars in the N-deficient treatment, indicating that maximum acclimation changes may happen at this scale. Interestingly, N_FR_ but not N_LR_ was significantly different between tolerant and sensitive cultivars. This may indicate that sampling of fine roots may be more important to understand differences in root N uptake. At leaf scale, gas exchange parameters such as R_d_, V_cmax_, and g_m_, although showed numerical difference, were not significantly different between the tolerant and sensitive cultivar. This may be associated with relatively large variability associated with these measurements. For the same reason, these measurements may not be most suitable for screening purposes. Among the root traits, only DW_LR_ was significant between the tolerant and sensitive cultivar in the N-deficient treatment. Given the difficulty associated with measuring root traits and lack of significance in DW_Root_, DW_FR_, and RWR between the sensitive and tolerant cultivars, these traits may be better suitable for understanding in-depth physiological mechanisms rather than screening purposes. No difference in SLA suggests proportionate changes in Area_Leaf_ and DW_Leaf_. These results clearly indicate that tolerance or sensitivity in the N-deficient treatment was regulated by selective traits.

**Table 4:** Traits at cellular, leaf, and whole-plant scales which were significantly different [increased (↑) or decreased (↓)] or non-significant between tolerant and sensitive cultivars in the N-deficient treatment. Refer to Tables 1–3 for trait names.

Scale	Significant	Non-Significant
Cellular	N_Leaf_ (**↑**) N_FR_ (**↑**) CHLD (**↑**) CTD (**↑**) ACN (↓)	N_Stem_ N_LR_
Leaf	Pg_max_ (**↑**) LSP (**↑**) J_max_ (**↑**)	R_d_ LCP LUE V_cmax_ g_m_
Whole-plant	DW_Shoot_ (**↑**) DW_Leaf_ (**↑**) Area_Leaf_ (**↑**) DW_LR_ (**↑**)	DW_Stem_ DW_Root_ DW_FR_ SLA RWR

The path analysis predicted three independent sub-paths relating traits at the cellular, leaf, and whole-plant scales (Fig 4). These models were associated with ACN, CHL, and P_gmax_. Interestingly, a single path model that includes all three sub-paths or any two sub-paths did not fit the data. This suggested that these three sub-paths were independent to each other.

Based on the ACN model, N_Leaf_ is positively correlated with N_FR_ and higher DW_Root_ (likely DW_LR_, Table 3). Further, the model predicts that N_Leaf_ negatively affected ACN levels, which further negatively affected Area_Leaf_ and DW_Shoot_ in the N-deficient treatment. Therefore, increased lateral root growth likely enabled fine root access to a larger substrate volume thereby leading to increased N_FR,_ higher N_Leaf_, and lower ACN in the tolerant cultivar. Further a lower ACN resulted in higher Area_Leaf_ and DW_Shoot_ in the tolerant than sensitive cultivar (Table 3). The model predicts that lower ACN in tolerant than sensitive cultivar is likely due to increased N_Leaf_ in the tolerant cultivar (Table 1). Anthocyanin accumulation is an important plant-adaptive response under N-deficient conditions [24–26]. Anthocyanin absorbs light in the blue and green wavebands and protects the subjacent chloroplasts from photoinhibition, especially when photosynthesis rate and light-use in photosynthesis are lower [27–29]. Therefore, ACN levels can increase in response to decreased levels of N_Leaf_. Moreover, a relatively large metabolic cost can be associated with increased biosynthesis of ACN in plants [30, 31], which involves the production of several enzymes and the transportation of ACN molecules into the vacuole. This process competes with the expansion growth for glucose produced in photosynthesis [32, 33]. Therefore, increased biosynthesis of ACN can reduce vegetative growth. This is the likely reason for a decrease in Area_Leaf_ in the sensitive than tolerant cultivar, in the N-deficient treatment. Finally, a larger Area_Leaf_ is associated with increased light interception leading to increased biomass production in plants [19].

The CHL sub-path model indicated a direct association between N_Leaf_ and CHL in plants. Further, the model predicted that CHL positively affected J_max_ in plants exposed to N-deficient conditions. In this model, J_max_ was positively correlated with DW_Leaf_, likely through photosynthesis rate in plants. Therefore, increased N_Leaf_ in the tolerant than sensitive cultivar (Table 1) resulted in higher CHL in the tolerant cultivar. Increased CHL and CTD with increasing N supply was reported earlier [34]. Both CHL and CTD are components of the light harvesting complex of photosynthesis [35, 36], and were previously associated with increased the efficiency of electron transport rate in plants [17]. Further, higher partitioning of N to the light harvesting complex of photosynthesis increased electron transport rate and photosynthesis [35, 36], likely by increasing CHL levels. As J_max_ and photosynthesis are positively correlated [22, 37], one of the likely mechanisms associated with higher DW_Leaf_ in the tolerant than sensitive cultivar was likely a higher photosynthesis rate.

The P_gmax_ sub-path model indicated that N_Leaf_ affected P_gmax_, which further affected DW_Shoot_. The model also predicted a positive relationship of P_gmax_ with DW_LR_ and N_FR_. According to this model, higher N_Leaf_ resulted in higher P_gmax_ leading to higher DW_Shoot_, DW_LR_, and N_FR_ in the tolerant compared to the sensitive cultivar when exposed to N-deficient conditions. A higher P_gmax_ has been earlier reported to be linked to N content in the leaf [38]. It is likely that the positive relation between N_Leaf_ and P_g,max_ is mediated by rubisco, as a significant amount of N_Leaf_ is partitioned to this enzyme [14] and rubisco capacity directly influences P_g,max_ at saturating light levels in plants [39]. Further, it was reported previously that an increased N content in the tissue increased the level and activity of rubisco [15, 16]. Interestingly, this model predicted a feedback loop between P_gmax_ and N_Leaf_ in plants mediated by DW_LR_ and N_FR_. A higher DW_LR_ in the tolerant cultivar likely enabled increased access to nutrients in the substrate, compared to the sensitive cultivar. This likely improved N uptake in the tolerant than sensitive cultivar. However, increased N_FR_, in addition to increased access to nutrients, likely requires higher N transporter activity in the fine roots. Increase in root NO_3_^-^ uptake due to higher activity of transporter system by enhancing plasma membrane H^+^ -ATPase activity has been reported earlier [40]. The increased transporter activity is an ‘active and energy-driven’ process [41]. The energy needed for the increased transporter activity in the fine root membranes likely requires support from increased photosynthesis and transport of sugars from the shoot to fine roots. Therefore, it is likely that the increased P_gmax_ in the tolerant cultivar not only resulted in increased shoot growth, but also provided sugars for increased lateral root growth and energy needed in active N transport, compared to the sensitive cultivar. The net result was an increased N_FR_ that further enhanced N_Leaf_ and in turn P_gmax_ in the tolerant cultivar.

Both CHL and P_gmax_ models were associated with DW_Leaf_. This is likely through an increased operating photosynthesis rate in the tolerant cultivar. Photosynthesis rate is co-limited by both rubisco efficiency and electron transport rate [22, 23, 42]. In the tolerant cultivar, both P_gmax_ (indicator of rubisco efficiency at saturating light) and J_max_ increased, therefore likely resulted in higher photosynthesis leading to increased growth. Previously, a higher P_gmax_ was reported to be associated with an increased photosynthesis and growth [39]. Surprisingly, we did not see a significant increase in V_cmax_, however there was a numerical increase in this trait (Table 2). Collectively, the developed models in our study indicate an interplay among traits at cellular, leaf, and whole-pant scales is required for tolerance to N-deficient conditions. It is likely that all three paths were operative in the tolerant cultivar leading to increased DW_Shoot_.

Among the traits, Area_Leaf_, CHL, J_max_, ACN, and N_Leaf_, can be screened rapidly and non-invasively using image-based systems. Generally, Area_Leaf_ can be measured with highest accuracy among other traits using image-based systems [43]. However, the error value associated with this trait in our path model was high for predicting DW_Shoot_ (Fig 4). Both CHL and J_max_ can be estimated rapidly and easily using hand held sensing devices (e.g., chlorophyll meters and fluorometers), however these devices are generally expensive. The estimation of ACN is possible using image-based systems [44]. Because both tolerant and sensitive cultivars showed an increase in ACN levels in the N-deficient treatment, ACN measurement may complicate the screening process without proper controls. Finally, N_Leaf_ appears to be most suitable trait for developing genetic markers in molecular breeding programs aimed at developing new varieties with N stress tolerance. The N_Leaf_ was associated with all three sub-path models and appears to connect root and shoot traits in our study making this a key trait influencing N stress tolerance in plants. The estimation of N_Leaf_ can be done reliably, easily, and rapidly on young and old leaves using low-cost handheld image-based sensors [45, 46]. Optimal values of N_Leaf_ can vary between 25 to 40 mg∙g^-1^ in plants [45]. This information can be used to assess the trait value in large number of samples using hand held image-based sensors for selection purposes. Genetic markers for N_Leaf_ can be developed by combining image-based N_Leaf_ estimations with high throughput genotyping assays.

## Conclusions

A key finding from our study is that plant tolerance to N stress likely involves multiple paths connecting traits at different scales. Further, our study identifies that leaf N content is a key trait connecting root N uptake with shoot metabolism, and enables tolerance to N stress in plants. Rapid screening for leaf N status is possible using image-based platforms. The implication of our findings is that genetic markers for leaf N content can be valuable in selecting elite breeding lines for developing new cultivars that can tolerate low N application rates in crop production.

## Supporting information

S1 Fig An illustration of ebb and flow fertigation system used to grow and impose nitrogen treatment to plants in the study. See ‘Fertigation System’ in the Materials and Methods section for additional details on construction and components.
(TIFF)

S1 Data (XLSX)
