# Supplementary figures and images for: Connecting the dots: Path model to identify key phenotypic traits for screening plants with tolerance to nitrogen deficiency
Source: PLoS One. 2023 Aug 18;18(8):e0288729. doi: 10.1371/journal.pone.0288729 (PMC10437786; doi:10.1371/journal.pone.0288729)

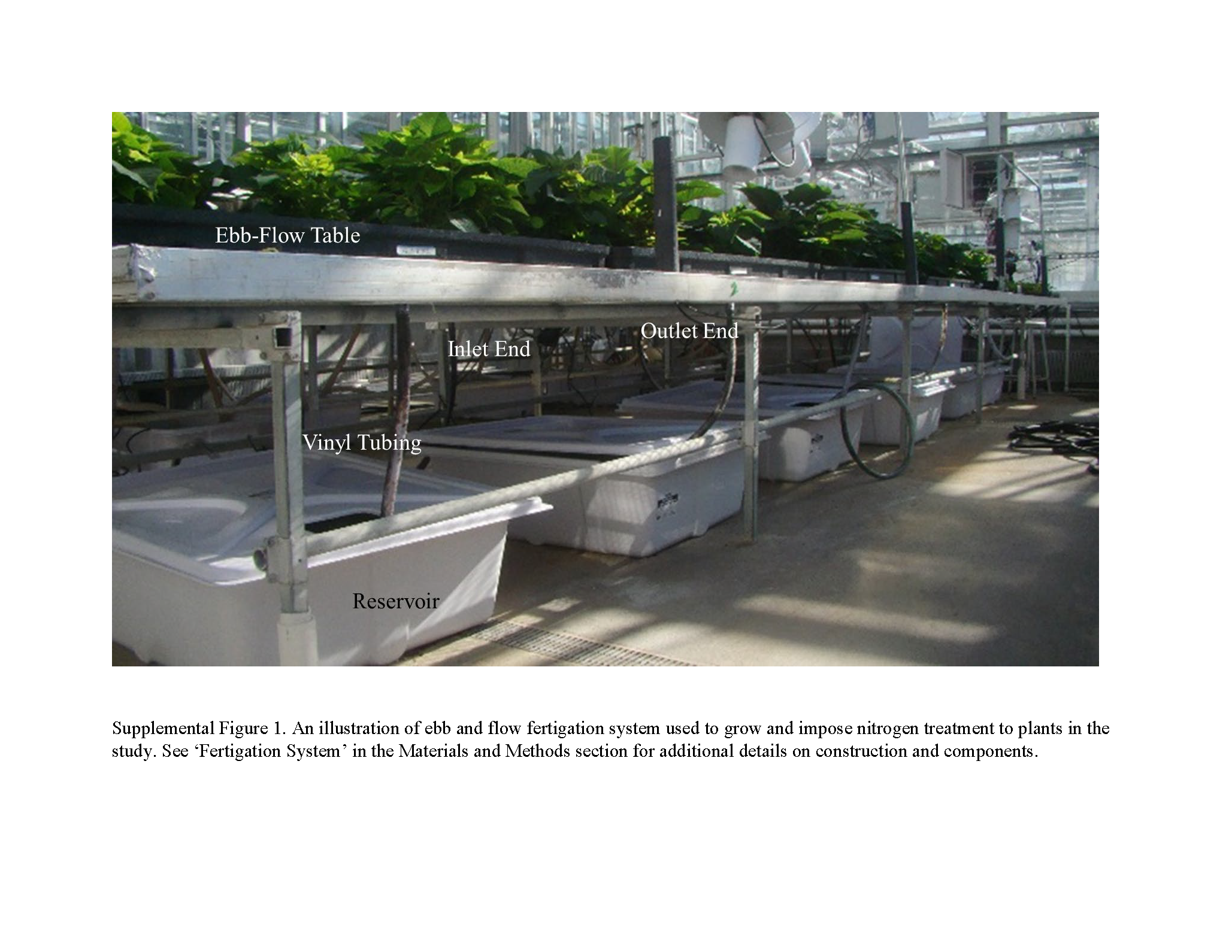

Supplement: S1 Fig — See ‘Fertigation System’ in the Materials and Methods section for additional details on construction and components. (TIFF) [file pone.0288729.s001.tiff]
